# Behavioral, cognitive, and socioemotional pathways from early childhood adversity to BMI: Evidence from two prospective, longitudinal studies

**DOI:** 10.1017/S0954579421001887

**Published:** 2022-05-12

**Authors:** Jenalee R. Doom, Ethan S. Young, Allison K. Farrell, Glenn I. Roisman, Jeffry A. Simpson

**Affiliations:** 1 Department of Psychology, University of Denver, Denver, CO, USA; 2 Department of Psychology, Utrecht University, Utrecht, The Netherlands; 3 Department of Psychology, Miami University, Oxford, OH, USA; 4 Institute of Child Development, University of Minnesota, Minneapolis, MN, USA; 5 Department of Psychology, University of Minnesota, Minneapolis, MN, USA

**Keywords:** adversity, BMI, early childhood, emotion dysregulation, impulsivity, overeating

## Abstract

Childhood adversity is associated with higher adult weight, but few investigations prospectively test mechanisms accounting for this association. Using two socioeconomically high-risk prospective longitudinal investigations, the Minnesota Longitudinal Study of Risk and Adaptation (MLSRA; *N* = 267; 45.3% female) and the Fragile Families and Child Wellbeing Study (FFCWS; *n* = 2,587; 48.5% female), pathways between childhood adversity and later body mass index (BMI) were tested using impulsivity, emotion dysregulation, and overeating as mediators. Childhood adversity from 0 to 5 years included four types of adversities: greater unpredictability, threat/abuse, deprivation/neglect, and low socioeconomic status. Parents reported on child impulsivity, emotion dysregulation, and overeating. Height and weight were self-reported and measured at 32 and 37 years in MLSRA and at 15 years in FFCWS. FFCWS results indicated that threat, deprivation, and low socioeconomic status predicted greater impulsivity and emotion dysregulation at 5 years, which in turn predicted greater overeating at 9 years and higher BMI *z*-score at 15 years. Early unpredictability in FFCWS predicted higher BMI through greater impulsivity but not emotion dysregulation at age 5. MLSRA regression results replicated the threat/abuse → emotion dysregulation → overeating → higher BMI pathway. These findings suggest that different dimensions of early adversity may follow both similar and unique pathways to predict BMI.

High rates of obesity in the United States and globally have led to growing interest in identifying precursors of obesity already present in childhood in order to identify potential early intervention targets. Existing research suggests that childhood adversity is consistently associated with overweight and obesity in adulthood (Danese & Tan, 2014; Farrell et al., 2017; Noll et al., 2007; Wells et al., 2010; Ziol-Guest et al., 2009), most likely due to a combination of cognitive, emotional, social, behavioral, and biological factors linking adversity to greater weight. Animal models similarly demonstrate that early life adversity causes greater body weight (Coccurello et al., 2009; Kaufman et al., 2007). Possible pathways explored in this literature include alterations in glucocorticoid regulation, metabolic and immune function, neurobiology, eating behaviors, coping strategies, and social behaviors.

However, existing work on the association between childhood adversity and body mass index (BMI) has some major limitations. Much of the work involves retrospectively reported experiences of childhood adversity, which can be problematic given difficulties with memory and reporting biases based on factors such as current health and psychopathology (Baldwin et al., 2019; Nivison et al., 2021). Although existing longitudinal studies provide consistent evidence of longitudinal associations between childhood adversity and adult weight gain, few prospective studies have tested pathways from childhood adversity to increased BMI over time (Midei & Matthews, 2011). The longitudinal studies of potential mechanisms that do exist typically cover shorter time periods ranging from months to years rather than decades. The current study addresses these limitations by testing pathways from childhood adversity to adolescent and adult BMI prospectively over 15–37-year intervals in two separate cohorts.

Although multiple types of adversity such as maltreatment, poverty, and social risk factors (e.g., violence exposure, separation from parents, and family turmoil) are associated with increased BMI over time, few studies have compared the effects of multiple types of adversity in the same investigation. Increasing theoretical work is being done to address how adversity can be partitioned into specific components that have unique influences on development (Epel et al., 2018; Humphreys & Zeanah, 2015; McLaughlin et al., 2014). For example, experiences can be broken down into dimensional measures of exposure to threat versus deprivation (Humphreys & Zeanah, 2015; McLaughlin et al., 2014). Models of early experiences inspired by evolutionary-developmental theory also suggest that experiences can be conceptualized as being high or low on harshness and/or unpredictability (Belsky et al., 2012; Brumbach et al., 2009; Ellis et al., 2009). It may be that certain types of adversity are more potent predictors of BMI or that different types of adversity have unique pathways to higher BMI, though these possibilities have rarely been investigated.

The current study investigates whether four different types of early adversity (low socioeconomic status [SES], high unpredictability, abuse/threat, and neglect/deprivation) are prospectively associated with pathways to higher BMI. The current study also investigates two potential pathways from early life adversity to higher adolescent and adult BMI (see conceptual model in Figure 1). The first proposed pathway investigates whether different types of early life adversities are associated with *greater impulsivity* in childhood, which then leads to greater overeating and higher BMI across time. The second pathway investigates whether different types of early life adversities are associated with *greater emotion dysregulation* in childhood, which then leads to greater overeating and higher BMI over time. Evidence for the validity of each of these potential mediators at least partially explaining the association between the four different types of early life adversity and later BMI is discussed below.

## Childhood adversity and impulsivity

Early childhood adversity, broadly defined, is associated with disruptions in cognitive functioning, including impulsivity. Exposure to *low SES* predicts greater impulsivity/less cognitive control in childhood (Evans & English, 2002; Noble et al., 2007).Additionally, *greater chaos and unpredictability* in childhood are related to greater impulsivity and problem behaviors associated with poor emotional and behavior regulation (Dumas et al., 2005; Evans et al., 2005). Greater home chaos is associated with greater delinquency, partially through its association with higher impulsivity (Joo & Lee, 2020).

Deficits in inhibition and cognitive control – correlates of impulsivity – have been reported for individuals with histories of experiencing trauma (Mueller et al., 2010; Navalta et al., 2006). Threat and deprivation appear to have independent associations with lower cognitive control. For example, both *childhood threat (abuse) and deprivation (neglect)* are associated with greater adolescent impulsivity (Oshri et al., 2018). Studies of institutionalized children whose experiences are often characterized by more severe physical and/or emotional neglect have documented poorer cognitive abilities, including problems with impulsivity, with the length of institutionalization being positively correlated with greater deficits (Loman et al., 2009; Pechtel & Pizzagalli, 2011; Rutter & O’Connor, 2004). Similarly, threat in the form of childhood abuse is associated with greater trait impulsivity in adulthood (Brodsky et al., 2001). When threat and deprivation are compared directly, cognitive deprivation/poverty appears to be more predictive of poorer cognitive control in adolescence than threat (Lambert et al., 2017).

Markers of chronic childhood adversity are also known to result in a behavioral phenotype of impulsivity, which mediates some of the link between adversity and poor health behaviors later in life (Miller et al., 2011). For example, recent work suggests that deficits in cognitive control may mediate the association between childhood trauma and greater waist circumference (Lunding et al., 2021). This phenotype of impulsivity may enhance fitness in harsh and/or unpredictable environments (Frankenhuis et al., 2016) by encouraging individuals to pursue rare or fleeting opportunities. One example is children from lower SES backgrounds eating more in the absence of hunger than children from higher SES backgrounds, which could protect them against later food scarcity (Hill et al., 2016). However, greater impulsivity is likely to have long-term costs to health, particularly in high-resource environments, given its association with unhealthy eating and higher BMI (Bénard et al., 2017; Jasinska et al., 2012; Schiff et al., 2016). Most of this prior research, however, is cross-sectional, and longitudinal research is needed to determine whether the impulsivity phenotype precedes poor health outcomes.

## Childhood adversity and emotion dysregulation

Early childhood adversity is reliably associated with greater difficulties in emotion regulation, which may also have important implications for health behaviors and BMI. *Lower SES* is related to a phenotype of poorer emotion regulation and more negative affect (Taylor et al., 2004), including differences in neural activation during emotion regulation tasks (Kim et al., 2013). Moreover, at least some of the effects of poverty on socioemotional functioning appear to be attributable to chaotic early living conditions (Evans et al., 2005), with *chaos/unpredictability* at home predicting greater negative emotionality in infants (Bridgett et al., 2013).

Finally, greater family adversity is known to be associated with greater emotion dysregulation in young children from disadvantaged backgrounds (Brown & Ackerman, 2011). In fact, nearly half of the effect of childhood adversity on recidivism in juvenile offenders is explained by their negative emotionality (Wolff & Baglivio, 2017). Childhood *threat (abuse)* is also associated with a phenotype of emotion regulation difficulties, including greater emotional instability (Stirling & Amaya-Jackson, 2008). Children who are exposed to early *deprivation/neglect* are also more likely to have a phenotype of greater emotion regulation difficulties, perhaps due to their heightened sensitivity to emotional situations (Pechtel & Pizzagalli, 2011; Tottenham et al., 2010). When compared directly, threat in the form of violence predicts poorer performance on tasks involving emotion regulation in adolescence compared to cognitive deprivation/poverty (Lambert et al., 2017). Although there is research demonstrating associations between different types of adversity and an emotion dysregulation phenotype, and between this phenotype and later health problems, there is little prospective data demonstrating associations over time.

It is important to note that impulsivity and emotion dysregulation are often correlated, with individuals demonstrating greater impulsivity being more likely to have problems with emotion regulation. Although correlation coefficients differ by population and the subtype of impulsivity and emotion dysregulation measured, coefficients have ranged from statistically non-significant to strong (e.g., *r* = .68) (Pivarunas & Conner, 2015; Weiss et al., 2015). A recent study of children and adolescents showed a moderate correlation of 0.36 (Heffer & Willoughby, 2021).

## Impulsivity and overeating

Impulsivity is one of neurocognitive domains that is most consistently associated with problematic eating behaviors and higher BMI (A. L. Miller, 2016; Vainik et al., 2013). Individuals with obesity are more likely to have high impulsivity, which often is a barrier to obesity treatment (Guerrieri et al., 2008; Nederkoorn et al., 2007). Greater trait impulsivity is also associated with greater food intake in healthy adults (Guerrieri et al., 2007). Specifically, attentional and motor impulsivity are strongly associated with binge eating and overweight (Meule & Platte, 2015). Furthermore, impulsivity predicts greater attentional bias to food cues, which can lead to greater consumption of high-calorie foods (Hou et al., 2011). In environments with many readily available foods, high-calorie food options, greater inattention, and impulsivity should be strongly associated with problematic eating behaviors and higher BMI (Davis, 2010).

## Emotion dysregulation and overeating

A growing literature has shown that poorer emotion regulation and negative affect may be links between early adversity and eventual obesity (Aparicio et al., 2016). Poorer emotion regulation, for example, is a good predictor of emotional overeating (Gianini et al., 2013), and individuals who have greater emotion regulation abilities report better ability to control overeating (Kerin et al., 2018). Negative affect and disordered eating have been implicated in the association between childhood adversity and greater adiposity (Midei & Matthews, 2011). Negative emotionality is a known risk factor for eating pathology and particularly bulimia (Keel & Forney, 2013; Peterson et al., 2010). Emotion dysregulation may increase the likelihood of overeating if food is used as a mechanism for coping with negative mood. Excessive food consumption, especially eating foods high in sugar, fat, or salt, may also be a form of self-medication or emotion regulation (Davis, 2010). Indeed, high-calorie diets tend to decrease physiological responsiveness to acute stress in animal models, supporting the notion of comfort food as a means of stress regulation (Morris et al., 2015). Thus, difficulty in regulating one’s emotions may lead to eating as a way to reduce negative emotions or regulate physiological responses to adversity.

## The current study

Our aim for the current research was to test whether greater impulsivity, emotion dysregulation, and overeating mediate associations between four types of early childhood adversity (low SES, high unpredictability, abuse/threat, and neglect/deprivation) and later BMI. Although these associations have been tested individually, to our knowledge, these pathways have not been tested together within prospective longitudinal cohorts. In the first cohort, the Fragile Families and Child Wellbeing Study (FFCWS), we test associations between adversity from 0–5 years and measured and self-reported BMI at 15 years, which is the latest wave of data collection. In the second cohort, the Minnesota Longitudinal Study of Risk and Adaptation (MLSRA), we test associations between adversity from 0 to 5 years and BMI in adulthood (self-reported at 32 and measured in the lab at 37 years). Specifically, we test whether certain types of early childhood adversity are more strongly associated with later BMI and attempt to determine whether specific types of adversity follow different pathways to higher BMI. In one of our samples (MLSRA), threat is measured as abuse, while in the other sample (FFCWS), threat is measured as parental harshness, including corporal punishment and psychological aggression. Similarly, in MLSRA, deprivation is measured as neglect, while in FFCWS, deprivation is measured as parental disengagement. Few studies have compared pathways to higher BMI from multiple types of adversity. However, there is evidence that threat may be more predictive of emotion dysregulation, and cognitive deprivation/poverty may be more predictive of impaired cognitive control (Lambert et al., 2017). As a result, we hypothesized that abuse/threat would predict a pathway from greater emotion dysregulation to overeating to higher BMI, and neglect/deprivation and low SES would predict a pathway from greater impulsivity to overeating to higher BMI.

## Methods

### Participants: FFCWS

The FFCWS (Reichman et al., 2001) is a population study of children born in large US cities. Participants were oversampled for non-marital births (74.9% of the current sample not married at their child’s birth). Most mothers had a high school degree (30.8%) or less than a high school degree (30.5%) at their child’s birth. Mothers averaged 25.2 years of age (SD = 6.0). FFCWS families were interviewed at the child’s birth and at 1, 3, 5, 9, and 15 years. Families were included if they provided self-reported (*n* = 2507) or measured (*n* = 888) height and weight data at 15 years and had valid data on covariates for inclusion in the final model.

The total number of participants who provided either self-reported or measured height and weight was 2587 (808 provided both, 1699 provided only self-reported, and 80 provided only measured height and weight). The current sample was 48.5% female. The self-reported racial/ethnic background of the sample was 49.5% Black non-Hispanic, 19.2% White non-Hispanic, 23.6% Hispanic/Latinx, 2.4% other non-Hispanic, and 5.3% multiracial non-Hispanic. There were no differences between the current and original samples by sex, maternal age, or marital status, all *p*s > .05, although mothers with lower education were less likely to be in the current sample compared to the original sample χ^2^ (3, *N* = 4892) = 50.0, *p* < .001. Table 1 shows correlations among the study variables.

### Participants: MLSRA

The MLSRA is a longitudinal study of the first-born children of mothers who were living below the poverty line when the study began from 1975 to 1977 (Sroufe et al., 2009). Mothers (*N* = 267, age range: 12–34 years, *M* = 20.5 years) were recruited during their third trimester. Most of the attrition occurred during the first two years of the study due to various reasons (usually moving away), but there has been modest attrition in the sample since then. A total of 220 participants (82.4% of the full sample) provided data on two or more early adversity indicators, age 5-year or 16-year mediators, or adult BMI. Participants with more complete data did not differ from the original sample by sex, birth weight, mother’s age at delivery, or maternal education, all *p*s > .05. In the full sample, 54.7% were male, and 45.3% were female, and 58.4% were White, 13.5% Black, and 16.1% Multiracial. A total of 2.6% identified as being another non-White identity, and 9.4% were listed as unknown due to incomplete information about the race of their father. Mother’s years of education at participant birth averaged 11.7 (SD = 1.8). The majority of mothers were single at their child’s birth (60.8%), with 35.5% married and 3.8% divorced, widowed, or separated. At age 32, 159 participants provided self-reported BMI (M = 28.5, SD = 7.3), and at age 37, 117 provided measured BMI (M = 30.3, SD = 7.5). Table 2 shows correlations between the study variables, and Table 3 compares the methods between the MLSRA and FFCWS cohorts.

### Psychosocial adversity: FFCWS

Psychosocial adversity (low SES, unpredictability, threat, and deprivation) was reported prospectively at birth and ages 1, 3, 5, 9, and 15 years. Mothers reported experiences at birth and 1 year. The primary caregiver reported experiences from 3 to 9 years. Both the primary caregiver and youth reported at 15 years. All variable names, questions, respondents, and response options from FFCWS are included in the supplemental methods, including correlations between measures of adversity at each timepoint.

#### SES

SES in FFCWS was calculated as a poverty ratio. From birth to 9 years, mothers reported on the household income and the number of family members in the home. Primary caregivers reported this information at 15 years. Poverty ratio variables at each timepoint from birth to 15 years were constructed by FFCWS. These variables were the household income divided by the US Census Bureau poverty threshold based on family composition and year, which were then *z*-scored within the FFCWS sample at each timepoint. The mean of these variables from birth to 5 years was used for the infancy and early childhood poverty variable, and the mean of these variables at 9 and 15 years was the middle childhood-to-adolescent poverty variable. Higher scores indicated higher SES. This composite SES variable was created for this study.

#### Unpredictability

Unpredictability in FFCWS was assessed by the following four measures of family instability: 1) changes in residence, 2) changes in parental cohabitation, 3) changes in employment, and 4) changes in father’s incarceration. Although father incarceration has not been previously included in composite measures of unpredictability, it represents an additional unique dimension of instability similar to changes in parental cohabitation where the father may be more present or absent at different points. Residential changes were determined by mothers reporting at all sessions between 1 and 15 years about the number of times they had moved since the last assessment. These variables were standardized within the sample at each wave. The means of the 1-year, 3-year, and 5-year variables were used as the changes in residence for the 0–5-year variable, and the means of the 9- and 15-year variables were used as the changes for the 5–15-year variable.

Mothers were asked about their relationship with their child’s biological father at each timepoint. If the mother reported being married or cohabiting with the father, they were assigned a 1; if they were not, they were given a 0. Changes in marriage/cohabitation from birth to 1 year, 1–3 years, 3–5 years, 5–9 years, and 9–15 years were given a 1, and no change was assigned a value of 0. The mean of the dichotomous variables from birth to 1 year, 1–3 years, and 3–5 years were used as the 0–5 year change in cohabitation variable, and the mean of the 5–9 years and 9–15 years were used as the 5–15 years variable.

Employment was assessed by asking mothers (or primary caregivers at 15 years) how many jobs they had for more than two weeks in the preceding 12 months. This question was asked at each wave from 5 to 15 years, and at 3 years, the mother was asked about the number of jobs since their child’s birth. Change in the number of jobs between each timepoint was calculated to create the following variables: change from 3 to 5 years, 5 to 9 years, and 9 to 15 years. These variables were standardized. The 3–5-years variable was used for the 0–5 years unpredictability variable to match the other 0–5 years variables, and the mean of the 5–9 years and 9–15 years variables was used for the 5–15 years employment change variable.

Mothers were asked at each timepoint whether their child’s biological father was currently in jail. If the biological father was in jail, they were given a score of 1 for that timepoint and a 0 if the father was not in jail. Changes in incarceration between each timepoint were given a value of 1 and no change given a value of 0, which resulted in the following five dichotomous variables: incarceration change from birth to 1 year, from 1 to 3 years, from 3 to 5 years, from 5 to 9 years, and from 9 to 15 years. The mean of the three dichotomous change variables from birth to 5 years was calculated as the change in incarceration variable from 0 to 5 years, and the mean of the two change variables from 5 to 15 years was calculated as the change in incarceration variable from 0 to 5 years. The 0–5 years and 5–15 years change in jail variables were standardized. The mean of the standardized changes in employment, residence, cohabitation, and jail variables from 0 to 5 years was calculated for the unpredictability from infancy and early childhood variable, and a parallel variable was calculated for middle childhood to adolescent unpredictability from 5 to 15 years. This variable was created for this study.

#### Threat

Variables measuring threat for ages 3, 5, and 9 were the same across the three waves though variables were different at 1 and 15 years. At 1 year and 15 years, variables were chosen to match the 3–9 years as much as possible. At 1 year, threat was measured as frequency of spanking by mother, father, and mother’s partner. Evidence from FFCWS suggests that spanking at 1 year predicts later child protective services involvement (Lee et al., 2014). Frequency of spanking was reverse coded, such that higher values indicate greater frequency. The variable was a sum of spanking frequency across the mother, father, and mother’s partner in the past month (0 = never spanked, 4 = spanked every day; possible range: 0–12). The sum was standardized for the 1 year threat variable. At 3, 5, and 9 years, a total of 10 items assessed threat frequency from the corporal punishment and psychological aggression subscales of the Conflict Tactics Scale (Straus et al., 1998), which includes items such as shaking, slapping, pinching, hitting, threatening, spanking, swearing at, and screaming at the child. Items were recoded with higher values indicating greater frequency of threat: 0 = never happened in the past year, 1 = once, 2 = twice, 3 = 3–5 times, 4 = 6–10 times, 5 = 11–20 times, 6 =>20 times. The 10 items were summed within the 3-, 5-, and 9-year timepoints and were then standardized. At 15 years, each youth was asked two questions from the conflict tactics scale: how often their primary caregiver: 1) shouts, yells, screams, swears, or curses at him or her; and 2) hits or slaps him or her. Replies were 1 = never, 2 = sometimes, and 3 = often. The primary caregiver was asked the same two questions about these actions towards their child with the same response options. The four variables were then standardized, and the mean was calculated for the 15 year threat variable. The infancy and early childhood threat variable was a mean of the standardized 1-, 3-, and 5-year threat variables, and the middle childhood to adolescent threat variable was a mean of the standardized 9- and 15-year threat variables. This variable has been used in a previous investigation (Doom et al., 2020).

#### Deprivation

Similar to the threat variables above, questions were the same at waves 3–9 years, and questions were chosen to assess parental deprivation at 1 year and 15 years as closely as possible. Deprivation at 1 year included seven variables chosen from the FFCWS questionnaires that were relevant to parental deprivation. Six of these variables have been used to measure maternal engagement (Turney & McLanahan, 2015), and one variable assessed lack of food for the child (a form of physical neglect): 1) how often the mother played games like “peek-a-boo” and “gotcha” with the focal child (recoded as 0 = every day, 1 = several times per week, 2 = several times per month, 3 = 1–2 times per month, 4 = never); 2) how often the mother sang songs or nursery rhymes to the child (coded as above); 3) how often the mother read stories to the child (coded as above); 4) how often the mother told stories to the child (coded as above); 5) how often the mother played inside with toys such as blocks or Legos with the child (coded as above); 6) how frequently the mother hugged or showed physical affection toward the focal child (coded as above); and 7) whether the child went hungry in the past year (coded into 0 = no, 1 = yes). The variables were standardized, and the mean was calculated for deprivation at 1 year.

At 3, 5, and 9 years, a total of five items measured frequency of deprivation experiences from the conflict tactics scale (Straus et al., 1998), including not being able to express their love to their child, leaving the child alone, not being able to give the child food or medical care, and being too drunk or high to care for their child. These items were re-coded with higher numbers indicating greater deprivation frequency: 0 = never happened in the past year, 1 = once, 2 = twice, 3 = 3–5 times, 4 = 6–10 times, 5 = 11–20 times, 6 = >20 times. The 10 items were summed at each timepoint, resulting in deprivation at 3, 5, and 9 years. These summary variables were then standardized.

Deprivation at 15 years was measured by five items (four reported by the primary caregiver, and one reported by the youth). These items were not part of an existing scale but were chosen because of their relevance to deprivation. Primary caregivers were asked: 1) how often drinking interfered with their responsibilities in past year; 2) how often the caregiver had problems with people because of drinking in past year; 3) how often illegal drug use interfered with responsibilities in past year; and 4) how often the caregiver had problems with people in the past year due to illegal drug use. Answers were coded as 0 = never, 1 = one time, or 2 = more than one time. Youths were asked how often they spend time alone in their home without an adult present, with responses coded as 0 = never, 1 = sometimes, 2 = often. Items were summed and then standardized for the 15 year deprivation variable.

The infancy and early childhood deprivation variable was a mean of the standardized 1-year, 3-year, and 5-year deprivation variables, and the middle childhood to adolescent deprivation variable was a mean of the standardized 9-year and 15-year deprivation variables. This conceptualization of deprivation is consistent with previous assessments, with physical neglect, emotional neglect, and food insecurity as part of the composite (Sumner et al., 2019). This variable has been used in a previous investigation (Doom et al., 2020).

### Psychosocial adversity: MLSRA

Psychosocial adversity in MLSRA was conceptualized as low SES, unpredictability, abuse, and neglect.

#### SES

Parents reported their SES just before the target child was born and when the child was 42 and 54 months old. An updated Duncan Socioeconomic Index (SEI; Stevens & Featherman, 1981) was used to assess occupational status. Occupational prestige was calculated based on education and income characteristics of people in the labor force in 1970. Household income and maternal education were also obtained. Information on each measure of SES (i.e., income, occupational status, and maternal education) was not collected at all timepoints. However, all three SES measures were collected at the prenatal assessment. SEI and maternal education were collected at 42 months, and SEI was also collected at 54 months.

To create a childhood SES composite, *z*-scores of all SES items within each time period were computed. The *z*-score values were transformed to *t*-scores (i.e., M = 50, SD = 10) to remove negative values. Lower scores indicated lower SES. Early SES was calculated by taking the mean of the SES *t*-scores within the age range of interest (ages 0–5). This measure has been used in past research with the MLSRA sample (Doom et al., 2016; Simpson et al., 2012).

#### Unpredictability

Unpredictability in the MLSRA was measured using mothers’ life adversity in three domains: 1) changes in residence, 2) changes in cohabitation, and 3) changes in employment. This unpredictability measure has been used in previous investigations with MLSRA data (Doom et al., 2016; Simpson et al., 2012; Szepsenwol et al., 2015). Information was obtained from the mother-reported life events schedule Egeland et al., 1980), which was measured five times in early childhood (when the child was 12, 18, 48, 54, and 64 months old). Coders rated the number of relevant events and the intensity of disruption that each one caused (0 = no disruption, 3 = severe disruption). All inter-rater reliabilities were greater than 0.90. Scores on the three measures were added within each time period. An average was then computed across the five early childhood timepoints (adjusted for the number of assessments the mother completed) to create a 0–5 year unpredictability score (M = 1.4, SD = 0.9, range: 0.0–5.2). Higher scores reflected greater exposure to unpredictability.

#### Threat and deprivation (abuse and neglect)

Information was prospectively collected about MLSRA participants’ experiences of physical abuse, sexual abuse, and neglect. Abuse and neglect coding criteria were based on Centers for Disease Control and Prevention (CDC) definitions. Physical abuse included a caregiver’s “intentional use of physical force against a child that results, or has the potential to result in, physical injury.” Sexual abuse was defined as sexual contact or noncontact exploitation (e.g., exposure of child to pornography) by a caregiver or by a perpetrator 5 or more years older than the child. Neglect was defined as the caregiver’s failure to provide for the child’s basic physical or cognitive needs, including hygiene, shelter, medical care, clothing, supervision, or education. The CDC definitions were supplemented by additional guidelines to identify abuse and neglect in the sample in consultation with MLSRA senior researchers, the available research literature, and Minnesota state law. The classifications of abuse or neglect are not necessarily those used as criteria by child protective services. Thus, our scoring does not indicate that child protective services were involved in the home.

The following coding system has been used in prior MLSRA studies (Labella et al., 2018). Participants whose records had been flagged during the study as potentially ever abused or neglected (*n* = 139, 52% of the sample) were examined more thoroughly. All available data collected from birth to 17.5 years were reviewed, which included information on caregiving quality, supervision, physical discipline, home environment, child protective service involvement, physical and sexual assault, and foster care history. This information was garnered from caregiver interviews, parent–child observations, reviews of child protection and medical records, teacher interviews, and adolescent reports. Reports of childhood abuse during the adult attachment interview, which was administered at 17.5 years of age, were not included unless abuse or neglect was previously identified, but there was not enough detail to code specifics about the developmental period.

Coding focused on whether physical abuse, sexual abuse, and/or neglect was present or absent within four developmental periods (infancy: 0–24 months; early childhood: 25 months to 5 years; middle childhood: 6–12 years; and adolescence: 13–17.5 years). Two coders reviewed every case and showed good to excellent reliability: Kappas ranged from .80 to .98 for presence or absence of physical abuse, sexual abuse, and/or neglect, and .80–.84 for presence or absence of each type during each development period. Any discrepancies were resolved by consensus. If physical abuse occurred during infancy, the child was assigned a 1; if it did not occur, the child was assigned a 0. This procedure was done for all four developmental periods and was also done for sexual abuse and neglect. Threat (abuse) from 0 to 5 years in this sample was the sum of physical and sexual abuse at infancy and early childhood (possible range: 0–4). Deprivation (neglect) from 0 to 5 years in this sample was the sum of neglect at infancy and early childhood (possible range: 0–2). Rates of abuse and neglect in MLSRA can be found in the supplemental materials.

#### Impulsivity: FFCWS and MLSRA

Impulsivity was measured in the same manner for the FFCWS and MLSRA datasets around age 5. Age 5 was chosen due to its temporal proximity to sensitive periods of early brain development during which adversity may impact developing cognitive and emotional systems (Zeanah et al., 2011). Impulsivity was measured with the child behavior checklist (CBCL), which asked parents whether their child is 1) impulsive or acts without thinking, 2) not able to concentrate or pay attention for long, and 3) not able to sit still or is restless. Parents responded on a 3-point scale (0 = not true, 1 = somewhat or sometimes true, 2 = very true or often true). These three items were averaged to create the impulsivity variable. Because the full CBCL was not administered in FFCWS due to time constraints, we used the questions that most closely mapped onto impulsivity and matched items administered in MLSRA. This variable was created for the purpose of this study. The mean in FFCWS was 0.5 (SD = 0.5; *α* = 0.64). The mean in MLSRA was 0.9 (SD = 0.5; *α* = 0.61). The differences between groups on mean impulsivity levels were statistically significant, *t* (2869) = 9.48, *p* < .001.

#### Emotion dysregulation: FFCWS and MLSRA

Emotion dysregulation was measured in the same manner for the FFCWS and MLSRA data sets around age 5. The CBCL asked parents whether their child 1) is stubborn, sullen, or irritable, 2) has sudden changes in mood or feelings, and 3) whether they have temper tantrums or a hot temper. Parents responded on a 3-point scale (0 = not true, 1 = somewhat or sometimes true, 2 = very true or often true). These three items were averaged to create the emotion dysregulation variable. This variable was created for the purpose of this study. The mean in FFCWS was 0.5 (SD = 0.5; *α* = 0.70). The mean in MLSRA was 0.7 (SD = 0.5; *α* = 0.62).

#### Overeating: FFCWS and MLSRA

Overeating was measured in the same manner for both data sets. Overeating was measured at age 9 in FFCWS and at age 16 in MLSRA. The CBCL overeating question asks parents to report whether their child eats too much on a 3-point scale (0 = not true, 1 = somewhat or sometimes true, 2 = very true or often true). The mean in FFCWS was 0.2 (SD = 0.5). The mean in MLSRA was 0.2 (SD = 0.5).

#### BMI: FFCWS and MLSRA

To ensure that results hold for both self-reported and measured height and weight, both were used in our analyses, though sample sizes were larger for self-reported height and weight in both studies, so these were used for the primary analyses. However, we used measured height and weight to confirm the results that used self-reported weight.

Height and weight in the FFCWS were self-reported for all participants (*n* = 2514 in the current sample) and measured on a subset of participants during a home visit at age 15 (*n* = 891 in the current sample). Height and weight were measured two times by the interviewer, and a third measurement was taken if the first measurements differed. For FFCWS, BMI was calculated and *z*-scored for age and sex using the CDC’s 2000 growth charts, which is recommended for use in children and adolescents (self-reported BMI *z*-score: M = 0.67, SD = 1.07; measured BMI *z*-score: M = 0.89, SD = 1.03). As CDC growth charts have a mean *z*-score of 0, the average BMI *z*-scores in this sample are above these mean values. One participant had a very low *z*-score (greater than 5 SDs from the mean), so this value was removed from our analyses.

Height and weight in MLSRA were self-reported at age 32 on the adult health survey (*n* = 159; M = 28.5, SD = 7.3) and measured in the lab at age 37 (*n* = 117; M = 30.3, SD = 7.5). The average BMI in the United States for women is 26.5, and for men it is 26.6, suggesting that the BMI of MLSRA participants was higher than the average American. For MLSRA, raw BMI was used as this measure is acceptable to use in adults.

### Data analysis

#### FFCWS

Longitudinal path analysis with bootstrapping (10,000 iterations) was conducted in Mplus 7.4 to estimate direct and indirect paths from unpredictability, deprivation, threat, and SES before age 5 to BMI at age 15. Given the nature of our sample, we cannot make claims about causal pathways, but rather test correlational paths over time. Two models were tested, which were the same except for the dependent variable. In the first model, the dependent variable was the self-reported BMI CDC *z*-score, and in the second model, the dependent variable was the measured BMI CDC *z*-score. Paths from early unpredictability, threat, deprivation, and SES to age 5 emotion dysregulation and impulsivity to age 9 overeating and then to age 15 BMI *z*-score were tested. We chose to use observed instead of latent variables because there were several variables in the model that were single items or measurements (e.g., BMI, overeating, unpredictability, SES, threat, and deprivation). In addition, there were some count variables (e.g., number of job changes or moves) and variables with restricted ranges (e.g., CBCL items), which led us to create composite variables rather than latent variables. Finally, it was unclear whether adversity was a reflective or formative construct, so observed variables were used.

The comparative fit index (CFI; >.93), root-mean-square error of approximation (RMSEA; <.06), standardized root-mean-square residual (SRMR; <.08) were examined to assess model fit (Hu & Bentler, 1999; Kline, 2015). The following variables were treated as covariates for impulsivity, emotion dysregulation, and overeating: sex and race/ethnicity. Race/ethnicity was dummy-coded as non-Hispanic White, Hispanic/Latinx, and other, with Black serving as the reference group as it was the largest racial group in FFCWS. The following variables were tested as covariates for BMI *z*-scores: sex, race/ethnicity, unpredictability from 9 to 15 years, deprivation from 9 to 15 years, threat from 9 to 15 years, and SES from 9 to 15 years. In the interest of model parsimony, a covariate was retained in the model only if it was associated with any of the variables at *p* < .05 (final covariates are shown in Table 4). Full information maximum likelihood (FIML) was used to estimate the models. Participants were included in the models if they provided self-reported (*n* = 2507) or measured (*n* = 888) height and weight data at 15 years and had valid data on covariates for inclusion in the final model.

#### MLSRA

The sample size of MLSRA was too small to conduct complex path analyses (Kline, 1998). Instead, we conducted regression analyses to replicate the results of the structural equation models we conducted in the much larger FFCWS sample. We chose to conduct these analyses in Mplus to use FIML in order to estimate the models given that some of the variables have missing data. These analyses extend the FFCWS analyses by examining overeating out to age 16 and BMI to age 32 and 37. Accordingly, we conducted five multiple regression models. These models examined the following five outcomes: emotion dysregulation at 5 years, impulsivity at 5 years, overeating at 16 years, BMI at 32 years, and BMI at 37 years. Predictors for the emotion dysregulation and impulsivity at 5 years in the models included unpredictability, deprivation/neglect, threat/abuse, and SES from infancy to early childhood, sex, and race/ethnicity (dummy coded as non-Hispanic/White or not non-Hispanic/White). Non-Hispanic/White served as the reference group as it was the largest racial/ethnic group in the MLSRA. Predictors for overeating at 16 years in the models included unpredictability, neglect, threat, and SES from infancy to early childhood, impulsivity at 5 years, emotion dysregulation at 5 years, sex, and race/ethnicity. Predictors for BMI at 32 years and 37 years in these models included unpredictability, neglect, threat, and SES from infancy to early childhood, impulsivity at 5 years, emotion dysregulation at 5 years, overeating at 16 years, sex, and race/ethnicity. Both self-reported and measured BMI at different ages were used because there were fewer participants with measured BMI, and we wanted to ensure associations were similar between self-reported and measured BMI in adulthood. The variances of all *x*-variables were mentioned in the MODEL command in Mplus to retain all cases, and FIML was used to handle missing data.

## Results

### FFCWS

**Figure 2.**
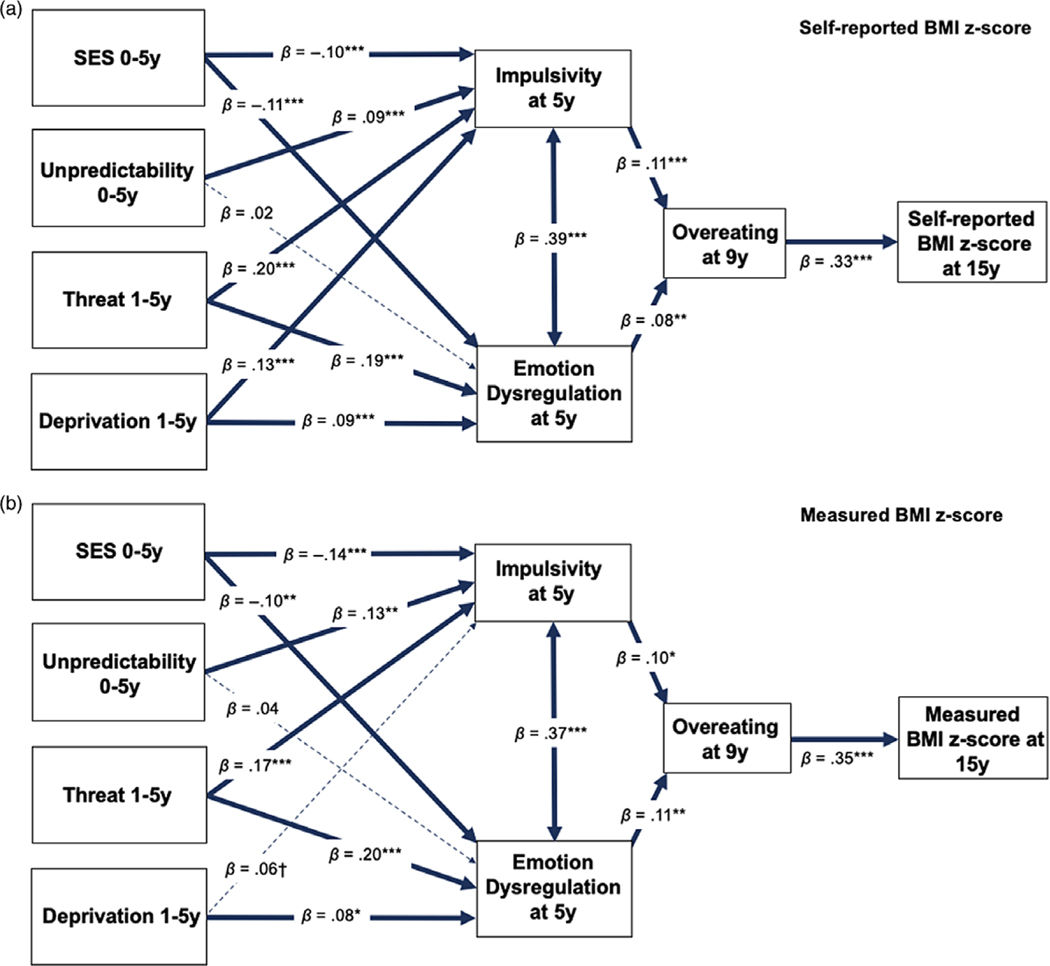
Early adversity to (a) self-reported and (b) measured BMI *z*-score at age 15 in FFCWS. Paths from early SES, unpredictability, abuse, and neglect to (a) self-reported and (b) measured BMI *z*-score at 15 years through emotion dysregulation, impulsivity, and overeating at 5 years and 9 years. Values are standardized coefficients. Solid blue lines represent significant pathways (*p* < .05) and dotted lines represent non-significant pathways. *p < .05, **p < .01, ***p < .001.

The self-reported BMI *z*-score model was saturated. The model revealed significant direct effects between impulsivity at 5 years and all forms of early adversity: SES, unpredictability, threat, and deprivation (see Table 4 and Figure 2a for direct effects). All of these early adversity exposures except for unpredictability were also associated with greater emotion dysregulation at age 5: SES, threat, and deprivation. Both impulsivity and emotion dysregulation predicted greater overeating at 9 years, and overeating at 9 years significantly predicted greater self-reported BMI *z*-score at age 15. SES, threat, and deprivation all showed indirect pathways to BMI *z*-scores through impulsivity and emotion dysregulation at 5 years and overeating at 9 years (see Table 5). Unpredictability had one indirect pathway to age 15 BMI *z*-scores through impulsivity at 5 years and overeating at 9 years. BMI *z*-score at 15 years showed direct associations with SES, *β* = −0.08, SE = 0.02, *p* < .001, and threat, *β* = 0.06, SE = 0.02, *p* = .005, but not with unpredictability or deprivation, *p*s > .05. The model explained 14.5% of the variance in self-reported BMI *z*-scores at age 15.

Results from the model with BMI *z*-scores measured at age 15 are shown in Figure 2b. The measured BMI *z*-score model was saturated. The model explained 14.7% of the variance in measured BMI *z*-score at 15 years. The model testing measured BMI *z*-scores largely replicated the findings in the self-reported BMI *z*-score model, especially in terms of the direction and magnitude of the direct effects (see Table 4 and Figure 2b). The two indirect pathways from threat to BMI *z*-score remained statistically significant, as did the path from SES → emotion dysregulation at age 5 → overeating at age 9 → BMI at age 15. However, some of the indirect pathways that were statistically significant in the self-reported model were not significant in the measured BMI model, most likely due to the sample decreasing to approximately one-third of the self-reported sample size or differences in the method of BMI measurement (see Table 5). Specifically, the indirect pathways from deprivation to measured BMI were no longer significant. The indirect pathways from SES → impulsivity at age 5 → overeating at age 9 → BMI at age 15 and the unpredictability → impulsivity at age 5 → overeating at age 9 → BMI at age 15 paths were also non-significant but marginally so, *p* < .10. Measured BMI *z*-score at 15 years showed direct associations with SES, *β* = −0.07, SE = 0.03, *p* = .012, and threat, *β* = 0.08, SE = 0.04, *p* = .017, but not with unpredictability or deprivation, *p*s > .05.

### MLSRA

The regression results for the MLSRA are shown in Table 6. Greater abuse (threat) from 0 to 5 years predicted greater emotion dysregulation and impulsivity at age 5, and greater unpredictability from 0 to 5 years predicted greater impulsivity at age 5. Greater emotion dysregulation at age 5 predicted greater overeating at age 16. Greater overeating at age 16 predicted greater BMI at ages 32 and 37, though greater emotion dysregulation at age 5 predicted lower BMI at age 37. Thus, there is evidence in the MLSRA that replicates the pathway from threat in infancy/early childhood to emotion dysregulation at age 5 to overeating at age 16 to greater BMI at ages 32 and 37. However, there is less evidence for other pathways, such as those from early SES and neglect predicting emotion dysregulation or impulsivity at age 5, or for impulsivity at age 5 predicting overeating at age 16.

## Discussion

The current study addresses the relative lack of research investigating pathways between types of early childhood adversity and later BMI using two prospective, longitudinal studies. With regard to FFCWS, the results demonstrate that each of the four types of early childhood adversity – low SES, unpredictability, threat, deprivation – have statistically significant indirect pathways to higher BMI years later. Lower SES and greater unpredictability, threat, and deprivation all predicted greater impulsivity at age 5, which in turn predicted more overeating and higher BMI. Lower SES and greater threat and deprivation – but not unpredictability – also predicted greater emotion dysregulation at age 5, which in turn predicted more overeating and higher BMI. The results from the MLSRA provide additional support for early threat/abuse predicting greater emotion dysregulation at age 5, emotion dysregulation at age 5 predicting overeating at age 16, and overeating at age 16 predicting greater BMI at ages 32 and 37. These findings advance our knowledge about how different types of early adversity may influence pathways leading to adolescent and adult health and inform interventions regarding how one might short-circuit these pathways to improve health.

### Different types of adversity

All four types of early adversity (low SES, unpredictability, threat, and deprivation) had statistically significant pathways to higher self-reported BMI at 15 years in FFCWS (Table 5). Except for early unpredictability, each type of adversity predicted pathways through both greater impulsivity and emotion dysregulation. However, early unpredictability predicted only a path through greater impulsivity to greater overeating and higher BMI. Findings from both cohorts suggest that early unpredictability may have stronger associations with the development of cognitive and behavioral regulation than with emotion regulation at age 5. This result was unexpected in view of mounting evidence that early unpredictability in parenting is associated with poorer cognitive *and* emotional development (Glynn & Baram, 2019). Our evidence suggests that associations between emotion dysregulation and lower SES, threat, and deprivation may be stronger than those between emotion dysregulation and early unpredictability. However, our measure of unpredictability includes events that may be more distal to the child (e.g., changes in parental employment) and more infrequent (e.g., moving) than measures of household chaos that have been used in the past to index unpredictability. It could be that this more distal measure of unpredictability has unique associations with impulsivity and emotion dysregulation compared to more proximal measures such as household chaos. More generally, this study demonstrates the unique predictive power of several different types of adversity for predicting pathways to higher BMI later in life. At the same time, it is important to point out that the magnitude of the *direct* associations between indicators of early adversity and BMI was modest, and these coefficients were not nominally significant in all cases.

Poverty and threat (often assessed as maltreatment) are commonly assessed in relation to BMI, and these previous studies often document positive relations between early adversity and BMI in adulthood. These different types of adversity, however, are rarely considered in the *same* study to determine whether there are *unique* associations between BMI and specific types of adversity or whether certain types of adversity are more potent predictors of BMI. In the current study, both lower SES and higher threat in infancy and early childhood revealed the most consistent associations with BMI later in life (i.e., statistically significant indirect paths replicated with measured BMI at 15 years), suggesting that these adversities may exert the strongest effects on adiposity. Low SES and experiences of abuse have been consistently associated with higher adiposity in high- and middle-income countries through neighborhood, psychological, and social factors, and our findings are consistent with this previous research (Claassen et al., 2019; Sacks et al., 2017).

Unpredictability is rarely assessed as a unique dimension of adversity, which could have implications for both mental and physical health. Unpredictability and chaos are unique constructs in their own right, not merely a proxy for other types of psychosocial adversity (Dumas et al., 2005). Although unpredictability does not appear to have effects on emotion dysregulation, it showed consistent associations with greater impulsivity in both cohorts. Early unpredictability may program brain development in ways that increase risk for later impulsive behaviors, which could have implications for health. Importantly, unpredictability predicted meaningful variance even beyond more well-studied adversities such as threat, deprivation, and lower SES. Thus, it is an important dimension of psychosocial adversity to consider when understanding the effects of adversity on development.

There is ongoing theoretical debate about whether adversity is best captured through specificity, cumulative, or dimensional models (McLaughlin et al., 2014; McLaughlin et al., 2021; Smith & Pollak, 2021). Although oversimplified here, some models of adversity suggest different exposures are uniquely associated with outcomes (e.g., dimensional models; McLaughlin et al., 2014), while other models suggest that exposures overlap and may have more similar than unique effects (Smith & Pollak, 2021). From our understanding of these models, we do not believe that our results provide support for one model over another as both suggest that there may be some similar and some unique pathways to various outcomes depending on the type of early adversity experienced. Further refinement of specific predictions from both models will allow for more direct comparisons of the models for different types of outcomes (e.g., types of psychopathology or physical health outcomes).

### Mediators

Previous studies have documented associations between certain types of early childhood adversity and impairments in behavior regulation. All four types of adversities were associated with greater impulsivity at age 5 in the FFCWS. However, only early abuse and unpredictability were associated with greater impulsivity at age 5 in the MLSRA. These non-significant associations may be attributable to low power. It is not surprising that these four types of adversities are associated with greater impulsivity in FFCWS given that global cognitive deficits may be a common phenotype following early-life adversity due to the protracted nature of brain development across childhood (Pechtel & Pizzagalli, 2011). It is interesting, however, that even after controlling for each of the types of adversity in the model, impulsivity still displayed unique associations with each type of adversity, demonstrating that each of these is important to consider in high-risk populations.

Prior research has also confirmed associations between early childhood adversity and emotion dysregulation. Consistent with these studies, threat, deprivation, and lower SES were all associated with emotion dysregulation in the FFCWS. Consistent with our hypotheses, abuse (threat) had the strongest associations with emotion dysregulation in the MLSRA. This finding is supported by research demonstrating that childhood abuse is associated with difficulties in emotion regulation, including greater emotional instability (Stirling & Amaya-Jackson, 2008). It is also consistent with research demonstrating that threat predicts poorer emotion regulation more strongly than cognitive deprivation/poverty (Lambert et al., 2017). Because these different types of adversities often co-occur, it is important to know which type(s) of adversity are uniquely predictive of impulsivity, emotion dysregulation, and pathways to higher BMI.

The findings of the current research support the hypothesis that early childhood adversity is prospectively related to a behavioral phenotype of impulsivity and emotion dysregulation and that these behavioral proclivities at least partially mediate the link between earlier adversity and poor adult health (Miller et al., 2011). This phenotype is characterized by immediate gratification and taking greater risks which may serve to enhance the possibility of reproducing in case of an early death (Figueredo et al., 2006; Nettle, 2010). This phenotype may also lead to greater impulsivity around food, especially if the availability of food in the future may be uncertain. Emotion dysregulation may also lead to overeating in the presence of highly palatable food or in stressful situations, potentially through increases in emotional eating or stress eating, eating to reduce feelings of negative emotion, or to dampen biological stress responses (Dallman, 2010; Dallman et al., 2003). Although these behavioral profiles may enhance fitness in the face of adversity and unpredictability, it may have costs with respect to long-term health (Frankenhuis et al., 2016). Given that most previous research has been cross-sectional, this study provides evidence that the phenotype of greater impulsivity and emotion dysregulation predicts overeating years later. Interventions designed to buffer the effects of adversity on cognitive, behavioral, and emotion regulation might, therefore, also improve physical health and reduce BMI.

The FFCWS confirmed the expected direction of associations between impulsivity, emotion dysregulation, and overeating, and the MLSRA confirmed the associations between emotion dysregulation and overeating. These associations are present after multiple years within each cohort, suggesting potential long-term processes leading to later BMI. Although overeating was measured in a more normative way, not examining specific disordered eating behaviors, we still observed these associations over time. Future research needs to determine whether and how disordered eating behaviors such as bingeing, in addition to more normative eating behaviors such as overeating or eating in the absence of hunger, mediate associations between early adversity and higher BMI (Mason et al., 2016). Currently, there is little research in this area, which is unfortunate given the urgent need to identify specific eating behaviors as intervention targets following childhood adversity (Mason et al., 2016).

When considering the downstream effects of adversity, we typically consider how difficulties in behavior and emotion regulation may influence psychopathology, but we less frequently consider their consequences for physical health. Many of the mediators between adversity and psychopathology may also mediate connections between adversity and poorer physical health. However, not all children who experience early adversity have disruptions in impulse control, emotion regulation, and eating behaviors and, as a result, the paths outlined in the current research are not likely to occur for every child who experiences early adversity. Indeed, there may be a subset who are susceptible to disruptions in behavior and emotion regulation or to overeating and higher BMI. Thus, it is important to consider which individuals are most likely to follow these pathways and to create tailored interventions for them. Possible sources of individual differences could include positive caregiving relationships or access to community resources or high-quality food (Campbell et al., 2014; Larsen et al., 2015; Schwarzenberg & Georgieff, 2018; Wilkinson et al., 2018). Potential individual genetic characteristics might also be investigated as sources of individual differences (Wickrama et al., 2014). In addition, we did not test all possible pathways from early adversity to later BMI in our analyses. Possible additional pathways to consider in future research would be alterations in social relationships, ongoing environmental adversity and food insecurity, and lack of medical or psychiatric care.

It is important to note that alterations in emotion regulation, impulsivity, and eating behaviors can also be viewed as adaptations to high-stress environments instead of dysregulation. These adaptations may be associated with poorer long-term physical health outcomes in part via increases in BMI. However, these same adaptations may help children to survive in a threatening, depriving, or unpredictable environment (Ellis et al., 2017; Frankenhuis et al., 2020; Mittal et al., 2015; Young et al., 2018). When resources are not consistently available, taking advantage of opportunities when they arise (e.g., overeating when food is available) may be a more effective and adaptive strategy than waiting.

### Strengths and limitations

The strengths of this study include the prospective measurement of adversity during the first 5 years of life, as well as prospective measures of impulsivity, emotion dysregulation, and overeating at later timepoints. Because most studies connecting these domains have been cross-sectional or retrospective in nature, the longitudinal prospective data in the current studies corroborate and expand upon these previous studies. In addition, it is quite rare in longitudinal studies to have such detailed measures of adversity exposure assessing threat, deprivation, SES, and unpredictability. These separate measures allowed us to tease apart the effects of each type of adversity in our samples. In addition, BMI was both measured and self-reported in each of the cohorts, allowing us to test whether associations were significant for both types of BMI data. Testing this difference is important because many studies have focused on only self-reported height and weight. Finally, the two-cohort design is another strength of the current research, allowing us to examine associations over time in the FFCWS and to confirm some associations within the smaller but longer-term MLSRA. Both of these cohorts are socioeconomically high-risk, which permitted us to explore severity and type of adversity better than we could have in a typical mid-to-high SES sample. It was especially encouraging that some of the results from the FFCWS, a sample largely composed of Black youth, replicated in the MLSRA, a sample largely composed of non-Latinx White participants, suggesting that pathways from adversity to BMI could be similar across race/ethnicity. Larger longitudinal studies are needed to test this possibility as dividing the sample further would decrease the power needed to test such complex models.

The limitations of the current investigation include having only reported and not observed measures of behavior, including impulsivity, emotion dysregulation, or overeating, all of which were reported by the mother or primary caregiver. The measures were collected at different timepoints, which lessens some of the concern that answers were attributable to a respondent’s emotions on a particular day. However, mothers and primary caregivers may have certain biases when reporting on their child’s behavior. Although we could have created composite measures of these behaviors using different questionnaires and reporters, we prioritized keeping the measures the same across the two cohorts. For this reason, the overeating measure was limited to a single item from the CBCL in both cohorts. We did not assess disordered eating in our overeating measure because we wanted to examine more normative rather than clinical eating behaviors, which were limited in both of our samples. Future research could incorporate disordered eating behaviors as mediators between adversity, cognition, emotion, and BMI. The internal reliability for our impulsivity and emotion dysregulation composites ranged from acceptable to good (*α* = .61–.70) (Ursachi et al., 2015), though variability in measurement could have led to variability in results across the cohorts. Other sample characteristics such as the significantly higher level of impulsivity reported in MLSRA could explain some of the differences in results between the cohorts. The current findings need to be replicated in other cohorts with behavioral measures of mediators or more detailed questions about overeating. We also acknowledge that BMI is just one measure of physical health, and future research should test whether similar pathways exist between different types of early adversity and physical health indicators. Finally, we were limited by a small sample size for the MLSRA, meaning we could not conduct the full model on this sample. However, due to the longer timeline of this study, we were able to examine associations with BMI at ages 32 and 37, which is much longer than the 15-year BMI assessment in the FFCWS. On the other hand, even though the FFCWS has not yet tracked its participants into adulthood, it is a much larger cohort, and we were able to effectively test our complex longitudinal model using the data.

## Conclusion

The current research adds to the literature by mapping longitudinal pathways from four unique dimensions of early childhood adversity – low SES, unpredictability, threat/abuse, deprivation/neglect – to BMI into adolescence and adulthood. The literature documenting how impulsivity, emotion dysregulation, and eating behaviors mediate associations between early adversity and BMI is growing, but it is still predominantly cross-sectional or retrospective in nature. Although neither of the cohorts we examined were designed to answer this particular research question, we were able to use these existing data sets to provide preliminary evidence for these associations across time using prospective longitudinal data, an improvement over previous cross-sectional or retrospective investigations. Future prospective longitudinal studies with both behavioral tasks as well as reports of behavior are needed to more fully understand how these pathways unfold across time en route to increasing BMI. This knowledge is needed to develop effective interventions that target specific types of early adversity to disrupt these pathways and improve health across development.

## Supplementary Material

1

## Figures and Tables

**Figure 1.**
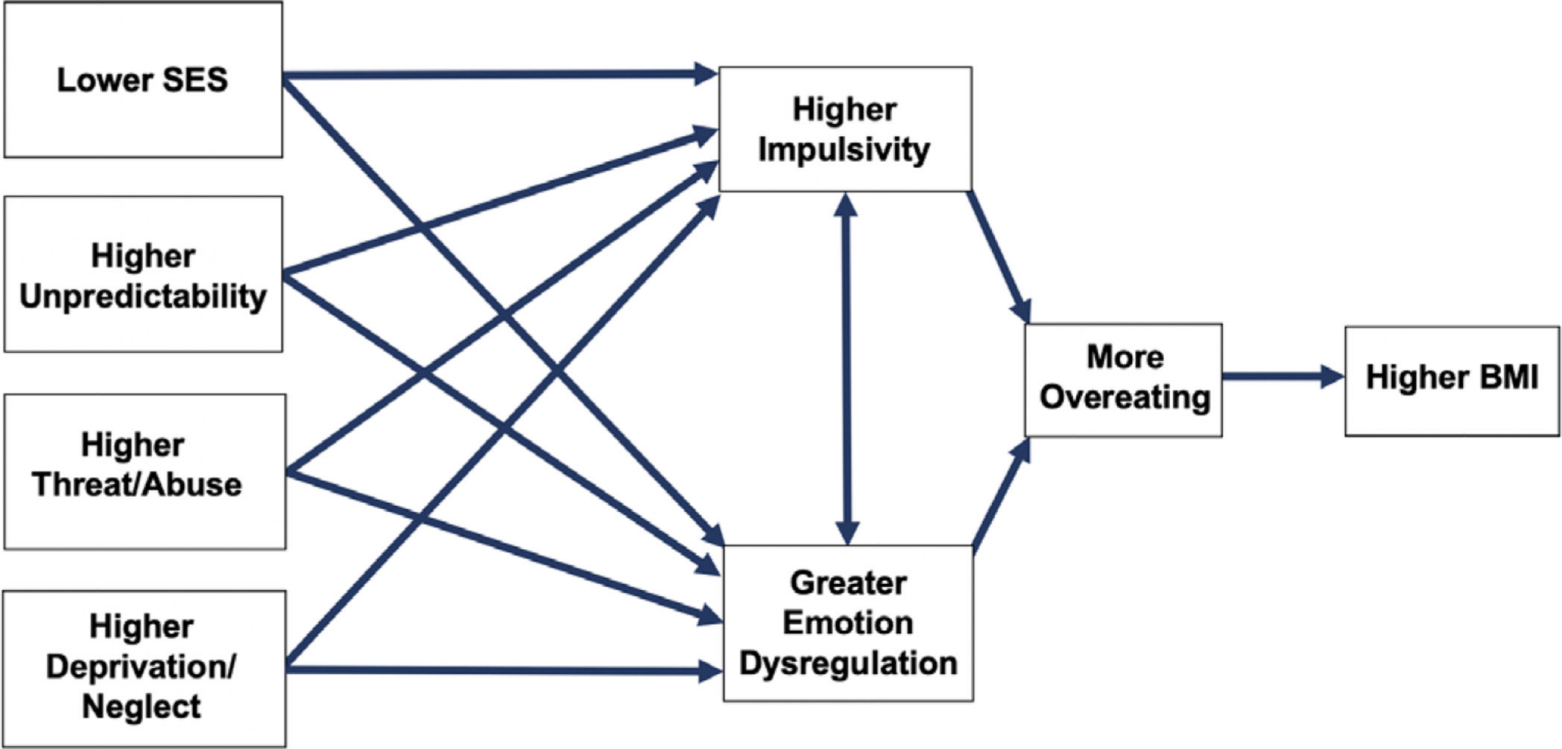
Conceptual model of mediators between socioeconomic status, unpredictability, threat/abuse, and deprivation/neglect to BMI.

**Table 1.** Correlations of main variables in FFCWS

Variable	1.	2.	3.	4.	5.	6.	7.	8.	9.	10.	11.	12.
1. SES 0–5 years	-											
2. Unpredictability 1–5 years	−.24***	-										
3. Threat 1–5 years	−.12***	.16***	-									
4. Deprivation 1–5 years	−.15***	.08***	.17***	-								
5. SES 9–15 years	.77***	−.20***	−.11***	− 13***	-							
6. Unpredictability 9–15 years	−.23***	.37***	.15***	.07**	−.24***	-						
7. Threat 9–15 years	− .10***	.15***	.43***	.10***	−.10***	.15***	-					
8. Deprivation 9–15 years	.03	.02	09***	.09***	.04*	.06**	.20***	-				
9. Impulsiveness at 5 years	−.11***	.13***	.22***	.17***	−.11***	.14***	.15***	.09***	-			
10. Emotion Dysregulation at 5 years	−.12***	.08***	.21***	.14***	−.12***	.13***	.19***	.04^†^	.43***	-		
11. Overeating at 9 years	−.06**	.01	.05*	.09***	−.05*	.04*	.12***	.08***	.15***	.13***	-	
12. Self-reported BMI *z*-score at 15 years	−.14***	.06**	.08***	.03	−.14***	.07**	.06**	.02	.07**	.04*	.34***	-
13. Measured BMI *z*-score at 15 years	−.10**	.06^†^	.08*	−.01	−.07*	.06*	.02	.01	.02	.01	.34***	.86***

*N* = 2587 (2507 with self-reported BMI and 888 with measured BMI). Correlations computed using SPSS v25.

† *p* < 0.10.

* *p* < .05.

** *p* < .01.

*** *p* < .001.

**Table 2.** Correlations of MLSRA variables

Variable	1.	2.	3.	4.	5.	6.	7.	8.	9.	10.	11.	12.
1. SES: Infancy to Early Childhood	-											
2. SES: Middle Childhood to Adolescence	.69***	-										
3. Unpredictability: Infancy to Early Childhood	−.18**	−.14*	-									
4. Unpredictability: Middle Childhood to Adolescence	−.22**	−.44***	.25***	-								
5. Abuse: Infancy to Early Childhood	−.08	−.17*	.22**	.12	-							
6. Abuse: Middle Childhood to Adolescence	−.19**	−.19*	.26***	.15*	42***	-						
7. Neglect: Infancy to Early Childhood	−.36***	−.34***	.28***	.26**	37***	.31***	-					
8. Neglect: Middle Childhood to Adolescence	−.19*	−.28***	.14^†^	.27***	.10	.20**	.42***	-				
9. Impulsiveness at 5 years	−.11	−.18*	.23**	.11	.26**	.23**	.22**	.09	-			
10. Emotion Dysregulation at 5 years	−.09	−.18*	.13^†^	.13^†^	.26**	.18*	.22**	.05	.41***	-		
11. Overeating at 16 years	.03	.06	.05	.11	−.01	.17*	−.04	.09	.10	.17*	-	
12. Self-reported BMI at 32 years	−.05	−.10	.19*	.15^†^	.16^†^	.16*	.13	.04	.17*	−.02	.41***	-
13. Measured BMI at 37 years	−.14	−.11	.04	.14	.00	.05	.00	.03	.01	−.24*	.31**	.76***

*n* = 266.

† *p* < 0.10.

* *p* < .05.

** *p* < .01.

*** *p* < .001.

**Table 3.** Comparison between FFCWS and MLSRA methods

Construct	*FFCWS*	*MLSRA*
SES	Poverty ratio (household income divided by poverty threshold for family size and year) from 0 to 5 years	Income, occupational status, and maternal education at various timepoints from 0 to 5 years
Unpredictability	Changes in residence, parental cohabitation, employment, or father’s incarceration from 0 to 5 years	Changes in residence, cohabitation, or employment from 0 to 5 years
Threat	Spanking at 1 year and harsh parenting tactics at 3–5 years	Abuse from 0 to 5 years
Deprivation	Measures of parental engagement and food access from 1 to 5 years	Neglect from 0 to 5 years
Impulsiveness	Parent-reported CBCL (three questions) at 5 years	Parent-reported CBCL (three questions) at 5 years
Emotion Dysregulation	Parent-reported CBCL (three questions) at 5 years	Parent-reported CBCL (three questions) at 5 years
Overeating	Parent-reported CBCL overeating question at 9 years	Parent-reported CBCL overeating question at 16 years
BMI	Self-reported at 15 years Measured at 15 years	Self-reported at 32 years Measured at 37 years

**Table 4.** Estimates of direct pathways from the predictor variables to impulsivity and emotion dysregulation at age 5, overeating at age 10, and self-reported BMI at age 15 in FFCWS

	Self-reported BMI model results (*n* = 2507)		Measured BMI model results (*n* = 888)	
	*β*	95% CI	*β*	95% CI
*Impulsivity at age 5*				
SES 0–5 years	−0.10***	−0.15, −0.06	−0.14***	−0.21, −0.06
Unpredictability 0–5 years	0 09***	0.05, 0.13	0.13**	0.05, 0.21
Threat 1–5 years	0 20***	0.16, 0.24	017***	0.11, 0.24
Deprivation 1–5 years	0.13***	0.09, 0.18	0.06†	−0.002, 0.13
Female	−0.12***	−0.16, −0.08	−0.15***	−0.21, −0.08
Race/Ethnicity: White	0.16***	0.11, 0.20	0.15***	0.08, 0.23
Race/Ethnicity: Latinx	0 09***	0.04, 0.13	0.13***	0.06, 0.20
Race/Ethnicity: Other	0.03	−0.01, 0.07	0.10**	0.03, 0.17
*Emotion dysregulation at age 5*				
SES 0–5 years	−0.11***	−0.15, −0.07	−0.10**	−0.17, −0.04
Unpredictability 0–5 years	0.02	−0.03, 0.07	0.04	−0.03, 0.12
Threat 1–5 years	019***	0.14, 0.23	0 20***	0.12, 0.27
Deprivation 1–5 years	0 09***	0.04, 0.14	0.08*	0.01, 0.15
Female	−0.02	−0.06, 0.02	−0.01	−0.08, 0.06
Race/Ethnicity: White	0.08***	0.04, 0.13	0.07^†^	−0.01, 0.14
Race/Ethnicity: Latinx	0.01	−0.04, 0.06	−0.02	−0.09, 0.06
Race/Ethnicity: Other	0.01	−0.03, 0.06	0.06	−0.01, 0.14
*Overeating at age 9*				
SES 0–5 years	−0.02	−0.06, 0.03	0.00	−0.07, 0.07
Unpredictability 0–5 years	−0.02	−0.07, 0.03	−0.04	−0.12, 0.05
Threat 1–5 years	0.00	−0.05, 0.05	0.01	−0.06, 0.08
Deprivation 1–5 years	0.06*	0.004, 0.11	0.03	−0.06, 0.10
Impulsivity at 5 years	011***	0.05, 0.16	0.10*	0.02, 0.18
Emotion Dysregulation at 5 years	0.08**	0.02, 0.13	0.11**	0.03, 0.19
Female	0.02	−0.02, 0.06	0.01	−0.06, 0.08
Race/Ethnicity: non-Latinx White	−0.04	−0.09, 0.01	−0.10**	−0.17, −0.03
Race/Ethnicity: Latinx	0.03	−0.02, 0.07	0.04	−0.04, 0.11
Race/Ethnicity: Other	0.01	−0.04, 0.06	0.03	−0.05, 0.10
*BMI z-score at age 15*				
SES 0–5 years	−0.08***	−0.12, −0.04	−0.07*	−0.13, −0.02
Unpredictability 0–5 years	0.02	−0.02, 0.06	0.04	−0.02, 0.11
Threat 1–5 years	0.06**	0.02, 0.10	0.08*	0.02, 0.15
Deprivation 1–5 years	−0.03^†^	−0.07, 0.002	−0.05	−0.11, 0.02
Impulsivity at 5 years	0.03	−0.02, 0.07	−0.02	−0.09, 0.06
Emotion Dysregulation at 5 years	−0.03	−0.07, 0.02	−0.06	−0.14, 0.01
Overeating at 9 years	0.33***	0.29, 0.37	0.35***	0.30, 0.41
Female	0.06**	0.02, 0.10	0.10**	0.04, 0.16
Race/Ethnicity: White	−0.08***	−0.13, −0.04	0.03	−0.04, 0.10
Race/Ethnicity: Latinx	0.00	−0.05, 0.04	0.03	−0.04, 0.10
Race/Ethnicity: Other	−0.02	−0.06, 0.01	−0.02	−0.08, 0.03

*Note.* Results on the left are for the self-reported BMI model (*n* = 2507), and results on the right are for the measured BMI model (*n* = 888). Reference group for Race/Ethnicity is Black.

† *p* < .10.

* *p* < .05.

** *p* < .01.

*** *p* < .001.

**Table 5.** Significant indirect pathways for self-reported and measured BMI in FFCWS

	*β*	95% CI
*Self-reported BMI at age 15 (n = 2,507)*		
SES 0–5 years → Impulsivity S years → Overeating at 9 years → BMI	−0.004**	−0.006, −0.001
SES 0–5 years → Emotion Dysregulation S years → Overeating at 9 years → BMI	−0.003*	−0.005, 0.000
Unpredictability 0–5 years → Impulsivity 5 years → Overeating at 9 years → BMI	0.003**	0.001, 0.005
Threat 1–5 years → Impulsivity 5 years → Overeating at 9 years → BMI	0.007**	0.003, 0.011
Threat 1–5 years → Emotion Dysregulation S years → Overeating at 9 years → BMI	0.005*	0.001, 0.008
Deprivation 1–5 years → Impulsivity 5 years → Overeating at 9 years → BMI	0.005**	0.002, 0.007
Deprivation 1–5 years → Emotion Dysregulation S years → Overeating at 9 years → BMI	0.002*	0.000, 0.004
*Measured BMI at age 15 (n = 888)*		
SES 0–5 years → Emotion Dysregulation 5 years → Overeating at 9 years → BMI	−0.004*	−0.008, 0.000
Threat 1–5 years → Impulsivity 5 years → Overeating at 9 years → BMI	0.006*	0.000, 0.012
Threat 1–5 years → Emotion Dysregulation 5 years → Overeating at 9 years → BMI	0.007*	0.001, 0.014

† *p* < .10.

* *p* < .05.

** *p* < .01.

*** *p* < .001.

**Table 6.** Regression results for MLSRA (*n* = 267)

	Estimate (*B*)	SE	95% CI	Est./SE	*p*-value
*Emotion Dysregulation at age 5*					
SES 0–5 years	−0.06	0.11	−0.28, 0.16	−0.52	.60
Unpredictability 0–5 years	0.03	0.09	−0.14, 0.20	0.35	.73
Abuse 0–5 years	0.20*	0.08	0.04, 0.35	2.44	.015
Neglect 0–5 years	0.14	0.09	−0.03, 0.32	1.61	.11
Female	0.10	0.15	−0.19, 0.39	0.68	.50
Race/Ethnicity: Not White/non-Hispanic	−0.23	0.15	−0.53, 0.07	−1.52	.13
*Impulsivity at age 5*					
SES 0–5 years	−0.06	0.11	−0.27, 0.16	−0.53	.60
Unpredictability 0–5 years	0.18*	0.08	0.01, 0.34	2.08	.037
Abuse 0–5 years	0.18*	0.08	0.02, 0.33	2.26	.024
Neglect 0–5 years	0.08	0.09	−0.09, 0.26	0.94	.35
Female	−0.10	0.15	−0.39, 0.19	−0.66	.51
Race/Ethnicity: Not White/non-Hispanic	0.08	0.15	−0.21, 0.38	0.54	.59
*Overeating at age 16*					
SES 0–5 years	0.05	0.10	−0.13, 0.24	0.57	.57
Unpredictability 0–5 years	0.06	0.09	−0.12, 0.23	0.64	.52
Abuse 0–5 years	−0.05	0.09	−0.22, 0.13	−0.53	.60
Neglect 0–5 years	−0.05	0.10	−0.25, 0.15	−0.46	.64
Emotion Dysregulation at 5 years	0.19*	0.09	0.02, 0.36	2.22	.027
Impulsivity at 5 years	0.02	0.09	−0.15, 0.19	0.25	.80
Female	−0.09	0.16	−0.40, 0.21	−0.59	.55
Race/Ethnicity: Not White/non-Hispanic	0.28^†^	0.16	−0.04, 0.60	1.72	.085
*Self-reported BMI z-score at age 32*					
SES 0–5 years	−0.03	0.09	−0.19, 0.14	−0.34	.74
Unpredictability 0–5 years	0.13	0.08	−0.03, 0.28	1.61	.11
Abuse 0–5 years	0.13	0.09	−0.04, 0.30	1.55	.12
Neglect 0–5 years	−0.01	0.10	−0.20, 0.18	−0.10	.92
Emotion Dysregulation at 5 years	−0.16^†^	0.08	−0.32, 0.001	−1.95	.05
Impulsivity at 5 years	0.06	0.08	−0.10, 0.23	0.73	.47
Overeating at 16 years	0.39***	0.07	0.25, 0.54	5.50	<.001
Female	−0.05	0.14	−0.32, 0.22	−0.35	.72
Race/Ethnicity: Not White/non-Hispanic	0.48**	0.15	0.19, 0.77	3.25	.001
*Measured BMI z-score at 37 years*					
SES 0–5 years	−0.21*	0.11	−0.42, −0.004	−2.00	.046
Unpredictability 0–5 years	0.03	0.10	−0.17, 0.23	0.26	.79
Abuse 0–5 years	0.04	0.11	−0.17, 0.25	0.37	.71
Neglect 0–5 years	−0.02	0.12	−0.25, 0.21	−0.17	.87
Emotion Dysregulation at 5 years	−0.28**	0.10	−0.48, −0.07	−2.68	.007
Impulsivity at 5 years	0.04	0.11	−0.17, 0.25	0.36	.72
Overeating at 16 years	0.32***	0.09	0.14, 0.50	3.53	<.001
Female	−0.24	0.17	−0.58, 0.09	−1.43	.15
Race/Ethnicity: Not White/non-Hispanic	0.29	0.18	−0.06, 0.64	1.62	.11

Note. Regression results for each of the five regression models in MLSRA with the dependent variable for each bolded.

† *p* < .10.

* *p* < .05.

** *p* < .01.

*** *p* < .001.
