# Supplementary material for: Behavioral, cognitive, and socioemotional pathways from early childhood adversity to BMI: Evidence from two prospective, longitudinal studies
Source: Dev Psychopathol. Author manuscript; Available in PMC 2023 Nov 1. (PMC9652481; doi:10.1017/S0954579421001887)
Supplement: 1 [file NIHMS1774333-supplement-1.docx]

***Supplemental Methods***

Sections within the supplement include: 1) Variables from FFCWS, and 2) Rates of abuse and neglect within MLSRA.

1. **Variables from FFCWS**

**SES**

| **Variable** | **Wave** | **Reporter** | **Question** | **Response** |
| --- | --- | --- | --- | --- |
| cm1inpov | Birth | Mother | Constructed: mother's household income/poverty threshold | [ratio] |
| cm2povco | 1y | Mother | Constructed: mother's household income/poverty threshold | [ratio] |
| cm3povco | 3y | Mother | Constructed: mother's household income/poverty threshold | [ratio] |
| cm4povco | 5y | Mother | Constructed: mother's household income/poverty threshold | [ratio] |
| cm5povco | 9y | Mother | Constructed: mother's household income/poverty threshold | [ratio] |
| cp6povco | 15y | Primary caregiver | Constructed: mother's household income/poverty threshold | [ratio] |

**Unpredictability**

| **Variable** | **Wave** | **Reporter** | **Question** | **Response** |
| --- | --- | --- | --- | --- |
| **Moving** |  |  |  |  |
| m2h1a | 1y | Mother | How many times have you moved since child's birth? | Participant responded with # of moves |
| m3i1a | 3y | Mother | How many times have you moved since child's first birthday? | Participant responded with # of moves |
| m4i1a | 5y | Mother | How many times have you moved in the last two years? | Participant responded with # of moves |
| m4f1a | 9y | Mother | How many times have you moved in the last 4 years? | Participant responded with # of moves |
| p6j2 | 15y | Primary caregiver | How many times have you moved since {Year 9 visit}? | Participant responded with # of moves |
| **Parent moving in and out** |  |  |  |  |
| cm1relf | Birth | Mother | Mother’s relationship with father | 1=married, 2 = romantic, cohabiting, 3 = romantic, visiting, 4 = friends, 5 = hardly talk, 6 = never talk, 7 = father unknown |
| cm2relf | 1y | Mother | Mother’s relationship with father | 1=married, 2 = romantic, cohabiting, 3 = romantic, some visiting, 4 = romantic, no visiting, 5 = separated/divorced/  widowed, 6 = friends, 7 = no relationship, 8 = father unknown |
| cm3relf | 3y | Mother | Mother’s relationship with father | 1=married, 2 = romantic, cohabiting, 3 = romantic, some visiting, 4 = romantic, no visiting, 5 = separated/divorced/  widowed, 6 = friends, 7 = no relationship, 8 = father unknown |
| cm4relf | 5y | Mother | Mother’s relationship with father | 1=married, 2 = romantic, cohabiting, 3 = romantic, some visiting, 4 = romantic, no visiting, 5 = separated/divorced/  widowed, 6 = friends, 7 = no relationship, 8 = father unknown |
| cm5relf | 9y | Mother | Mother’s relationship with father | 1=married, 2 = romantic, cohabiting, 3 = romantic, no visiting, 5 = separated/divorced/  widowed, 6 = friends, 7 = no relationship, 8 = father unknown |
| cp6mrelf | 15y | Primary caregiver | Biological primary caregiver’s relationship with biological parent | 1=married, 2 = romantic, cohabiting, 3 = romantic, no visiting, 5 = separated/divorced/  widowed, 6 = friends, 7 = no relationship, 8 = father unknown |
| **Job changes** |  |  |  |  |
| m3k22 | 3y | Mother | In the last 3 years, how many regular jobs have you had that lasted 2 weeks or more? | Participant responded with # of jobs |
| m4k22 | 5y | Mother | In the past twelve months, how many regular jobs have you had that lasted two weeks or more? | Participant responded with # of jobs |
| m5i22 | 9y | Mother | In the past twelve months, how many regular jobs have you had that lasted two weeks or more? | Participant responded with # of jobs |
| p6k34 | 15y | Primary caregiver | In the past twelve months, how many regular jobs have you had that lasted two weeks or more? | Participant responded with # of jobs |
| **Father in Jail** |  |  |  |  |
| cm1finjail | Birth | Mother | Constructed: mother report, father in jail at mother baseline interview | 1= yes, 0 = no |
| cm2finjail | 1y | Mother | Constructed: mother report, father in jail at mother 1y interview | 1= yes, 0 = no |
| cm3finjail | 3y | Mother | Constructed: mother report, father in jail at mother 3y interview | 1= yes, 0 = no |
| cm4finjail | 5y | Mother | Constructed: mother report, father in jail at mother 5y interview | 1= yes, 0 = no |
| cm5finjail | 9y | Mother | Constructed: mother report, father in jail at mother 9y interview | 1= yes, 0 = no |
| p6g24 | 15y | Primary caregiver | Has {BIOFATHER/CURRENT PARTNER} spent any time in jail or prison since {Year 9 visit}? | 1 = yes, 2 = no |
| p6f35 | 15y | Primary caregiver | Has {non-resident parent} spent any time in jail or prison since {Year 9 visit}? | 1 = yes, 2 = no |

**Threat**

| **Variable** | **Reporter** | **Question** | **Response** |
| --- | --- | --- | --- |
| **1y** |  |  |  |
| m2e5 | Mother | In the past month, has (CURRENT PARTNER) spanked (CHILD) because (he/she) was misbehaving or acting up? | 1 = yes; 2 = no |
| m2e5a | Mother | Did (CURRENT PARTNER) do this … | 1 = every day; 2 = a few/week; 3 = a few/month; 4 = once or twice |
| m2c4 | Mother | In the past month, has (FATHER) spanked (CHILD) because (he/she) was misbehaving or acting up? | 1 = yes; 2 = no |
| m2c4a | Mother | Did he do this (spank child) … | 1 = every day; 2 = a few/week; 3 = a few/month; 4 = once or twice |
| m2b19 | Mother | In the past month, have you spanked (CHILD) because (he/she) was misbehaving or acting up? | 1 = yes; 2 = no |
| m2b19a | Mother | Did you do this (spank CHILD) ... | 1 = every day; 2 = a few/week; 3 = a few/month; 4 = once or twice |
| **3y, 5y, 9y** |  |  |  |
| p3j3, p4g3, p5q1c* | Primary caregiver | How many times in the past year did you shake child? | 0 = never happened; 1 = once; 2 = twice; 3 = 3-5 times; 4 = 6-10 times; 5 = 11-20 times; 6 = > 20 times; 7 = yes, but not in past year |
| p3j4, p4g4, p5q1d* | Primary caregiver | How many times in the past year did you it (him/her) on the bottom with something like a belt, hairbrush, a stick or some other hard object? | 0 = never happened; 1 = once; 2 = twice; 3 = 3-5 times; 4 = 6-10 times; 5 = 11-20 times; 6 = > 20 times; 7 = yes, but not in past year |
| p3j6, p4g6, p5q1f* | Primary caregiver | How many times in the past year did you shout, yell, or scream at (CHILD)? | 0 = never happened; 1 = once; 2 = twice; 3 = 3-5 times; 4 = 6-10 times; 5 = 11-20 times; 6 = > 20 times; 7 = yes, but not in past year |
| p3j7, p4g7, p5q1g* | Primary caregiver | How many times in the past year did you spank (him/her) on the bottom with your bare hand? | 0 = never happened; 1 = once; 2 = twice; 3 = 3-5 times; 4 = 6-10 times; 5 = 11-20 times; 6 = > 20 times; 7 = yes, but not in past year |
| p3j8, p4g8, p5q1h* | Primary caregiver | How many times in the past year did you swear or curse at (him/her)? | 0 = never happened; 1 = once; 2 = twice; 3 = 3-5 times; 4 = 6-10 times; 5 = 11-20 times; 6 = > 20 times; 7 = yes, but not in past year |
| p3j9, p4g9, p5q1i* | Primary caregiver | How many times in the past year did you say you would send (him/her) away or would kick (him/her) out of the house? | 0 = never happened; 1 = once; 2 = twice; 3 = 3-5 times; 4 = 6-10 times; 5 = 11-20 times; 6 = > 20 times; 7 = yes, but not in past year |
| p3j10, p4g10, p5q1j* | Primary caregiver | How many times in the past year did you threaten to spank or hit (him/her) but did not actually do it? | 0 = never happened; 1 = once; 2 = twice; 3 = 3-5 times; 4 = 6-10 times; 5 = 11-20 times; 6 = > 20 times; 7 = yes, but not in past year |
| p3j11, p4g11, p5q1k* | Primary caregiver | How many times in the past year did you slap (him/her) on the hand, arm, or leg? | 0 = never happened; 1 = once; 2 = twice; 3 = 3-5 times; 4 = 6-10 times; 5 = 11-20 times; 6 = > 20 times; 7 = yes, but not in past year |
| p3j13, p4g13, p5q1m* | Primary caregiver | How many times in the past year did you pinch (him/her)? | 0 = never happened; 1 = once; 2 = twice; 3 = 3-5 times; 4 = 6-10 times; 5 = 11-20 times; 6 = > 20 times; 7 = yes, but not in past year |
| p3j14, p4g14, p5q1n* | Primary caregiver | How many times in the past year did you call (him/her) dumb or lazy or some other name like that? | 0 = never happened; 1 = once; 2 = twice; 3 = 3-5 times; 4 = 6-10 times; 5 = 11-20 times; 6 = > 20 times; 7 = yes, but not in past year |
| **15y** |  |  |  |
| k6c9c | Child | How often does your primary caregiver shout, yell, scream, swear or curse at you? | 1 = never, 2 = sometimes, 3 = often |
| k6c9d | Child | How often does your primary caregiver hit or slap you? | 1 = never, 2 = sometimes, 3 = often |
| p6d28 | Primary caregiver | How often in the past year have you shouted, yelled, screamed, swore or cursed at (him/her)? | 1 = never, 2 = sometimes, 3 = often |
| P6d29 | Primary caregiver | How often in the past year have you hit or slapped (him/her)? | 1 = never, 2 = sometimes, 3 = often |

*9y variables and responses were the same as 3y and 5y but had different coding (all were recoded before analysis.

**Deprivation**

| **Variable** | **Reporter** | **Question** | **Response** |
| --- | --- | --- | --- |
| **1y** |  |  |  |
| m2b18a | Mother | (How often do you) play games like "peek-a-boo" or "gotcha" with (CHILD)? | 205 = never; 204 = 1-2 times/month; 203 = several times/month; 202 = several times/week; 201 = every day |
| m2b18b | Mother | (How often do you) sing songs or nursery rhymes to (CHILD)? | 205 = never; 204 = 1-2 times/month; 203 = several times/month; 202 = several times/week; 201 = every day |
| m2b18c | Mother | (How often do you) read stories to (CHILD)? | 205 = never; 204 = 1-2 times/month; 203 = several times/month; 202 = several times/week; 201 = every day |
| m2b18d | Mother | (How often do you) tell stories to (CHILD)? | 205 = never; 204 = 1-2 times/month; 203 = several times/month; 202 = several times/week; 201 = every day |
| m2b18e | Mother | (How often do you) play inside with toys such as blocks or Legos with (CHILD)? | 205 = never; 204 = 1-2 times/month; 203 = several times/month; 202 = several times/week; 201 = every day |
| m2b18g | Mother | (How often do you) pug or show physical affection to (CHILD)? | 205 = never; 204 = 1-2 times/month; 203 = several times/month; 202 = several times/week; 201 = every day |
| m2h19b | Mother | In the past year, did your child go hungry? | 1 = yes, 2 = no |
| **3y, 5y, 9y** |  |  |  |
| p3j15, p4g15, p5q2a* | Primary caregiver | How many times in the past year did you have to leave your child home alone, even when you thought some adult should be with (him/her)? | 0 = never happened; 1 = once; 2 = twice; 3 = 3-5 times; 4 = 6-10 times; 5 = 11-20 times; 6 = > 20 times; 7 = yes, but not in past year |
| p3j16, p4g16, p5q2b* | Primary caregiver | How many times in the past year were you not able to show or tell your child that you loved (him/her)? | 0 = never happened; 1 = once; 2 = twice; 3 = 3-5 times; 4 = 6-10 times; 5 = 11-20 times; 6 = > 20 times; 7 = yes, but not in past year |
| p3j17, p4g17, p5q2c* | Primary caregiver | How many times in the past year were you not able to make sure (CHILD) got the food (he/she) needed? | 0 = never happened; 1 = once; 2 = twice; 3 = 3-5 times; 4 = 6-10 times; 5 = 11-20 times; 6 = > 20 times; 7 = yes, but not in past year |
| p3j18, p4g18, p5q2d* | Primary caregiver | How many times in the past year were you not able to make sure your child got to a doctor or hospital when (he/she) needed it? | 0 = never happened; 1 = once; 2 = twice; 3 = 3-5 times; 4 = 6-10 times; 5 = 11-20 times; 6 = > 20 times; 7 = yes, but not in past year |
| p3j19, p4g19, p5q2e* | Primary caregiver | How many times in the past year were you so drunk or high that you had a problem taking care of your child? | 0 = never happened; 1 = once; 2 = twice; 3 = 3-5 times; 4 = 6-10 times; 5 = 11-20 times; 6 = > 20 times; 7 = yes, but not in past year |
| **15y** |  |  |  |
| p6h82 | Primary caregiver | How often has your drinking interfered with your responsibilities at work or school, or at home in the past year? | 0 = never, 1 = 1 time, 2 = more than 1 time |
| p6h84 | Primary caregiver | How often have you had problems with your family, friends, or people at work or school because of your drinking in the past year? | 0 = never, 1 = 1 time, 2 = more than 1 time |
| p6h90 | Primary caregiver | How often has your illegal drug use interfered with your responsibilities at work or school, or at home in the past year? | 1 = never, 2 = 1 time, 3 = more than 1 time |
| p6h92 | Primary caregiver | How often have you had problems with your family, friends, or people at work or school because of your illegal drug use in the past year? | 1 = never, 2 = 1 time, 3 = more than 1 time |
| k6c8 | Child | How often do you spend time alone in your home without an adult present? | 1 = often, 2 = sometimes, 3 = never |

*9y variables and responses were the same as 3y and 5y but had different coding (all were recoded before analysis.

1. Rates of adversity in FFCWS

The following descriptive statistics are from the subset of FFCWS included in the current analyses (n = 2,587). The income/poverty ratio averaged 2.35 (SD = 2.50) at birth, 1.91 (SD = 2.32) at 1 year, 2.02 (SD = 2.76) at 3 years, 2.02 (SD = 2.37) at 5 years, 2.10 (SD = 2.40) at 9 years, and 2.43 (SD = 2.51) at 15 years.

The number of moves averaged 0.63 (SD = 0.88) from birth to 1 year, 0.67 (SD = 0.93) from 1 to 3 years, 0.70 (SD = 0.90) from 3 to 5 years, 1.07 (SD = 1.28) from 5 to 9 years, and 1.30 (SD = 1.52) from 9 to 15 years. A total of 21.2% of participants had a change in parental cohabitation from birth to 1 year, 15.8% from 1 to 3 years, 12.6% from 3 to 5 years, 13.0% from 5 to 9 years, and 11.3% from 9 to 15 years. The number of job changes between study visits averaged 0.88 (SD = 1.47; range: 0-28) between ages 3 and 5 years, 0.55 (SD = 1.15; range: 0-29) between ages 5 and 9, and 0.52 (SD = 0.87; range: 0-25) between ages 9 and 15. A total of 5.1% had a change in father’s incarceration from birth to 1 year, 5.3% from 1 to 3 years, 5.9% from 3 to 5 years, 7.5% from 5 to 9 years, and 17.0% from 9 to 15 years.

The spanking measure at 1 year ranged from 0-4 for mothers (M = 0.49, SD = 0.93), fathers (M = 0.19, SD = 0.59), and partners (M = 0.01, SD = 0.13). The mean of the ten threat items averaged 14.53 (SD = 8.95; range: 0-48) at age 3 years, 14.40 (SD = 8.93; range: 0-49) at 5 years, and 10.69 (SD = 8.43; range: 0-49) at age 9 years. At age 15 years, 65.7% of youth reported their primary caregiver shouted, yelled, screamed, swore, or cursed at them *sometimes or often*, and 12.7% reported their caregiver hit or slapped them sometimes or often. At 15 years, 69.7% of parents reported shouting or swearing at their youth *sometimes or often* in the past year, and 13.0% report hitting or slapping them sometimes or often in the past year.

These percent of mothers report doing these activities less than several times per week at age 1 year: 1) play games like peek-a-boo and gotcha (4.4%), 2) sing songs or nursery rhymes to child (8.9%), 3) how often the mother read stories to the child (24.6%); 4) how often the mother told stories to the child (33.4%); 5) how often the mother played inside with toys such as blocks or Legos with the child (6.8%); 6) how frequently the mother hugged or showed physical affection toward the focal child (0.4%). Only 0.7% of mothers reported that the focal child went hungry in the past year at the 1-year assessment. The mean of the ten deprivation items averaged 0.36 (SD = 1.39; range: 0-30) at age 3 years, 0.32 (SD = 1.14; range: 0-14) at 5 years, and 0.70 (SD = 1.80; range: 0-24) at age 9 years. At age 15, 0.6% of primary caregivers reported that drinking interfered with their responsibilities in past year, 0.4% had problems with people because of drinking in past year; 0.4% reported that illegal drug use interfered with responsibilities in past year; and 0.3% reported problems with people in the past year due to illegal drug use.

1. Rates of abuse and neglect within MLSRA

Within the current sample, 38.2% of individuals had ever experienced physical abuse, sexual abuse, or neglect (30.3% had not experienced abuse or neglect; 31.5% had missing data and could not be determined). A total of 14.2% experienced abuse from 0-5y (52.1% no abuse; 33.7% missing), 19.8% experienced neglect from 0-5y (47.6% no neglect; 32.6% missing), 21.0% experienced abuse from 6-17.5y (46.1% no abuse; 33.0% missing), and 12.8% experienced neglect from 6-17.5y (54.3% no neglect; 33.0% missing)*.*
